# Paired motor cortex and cervical epidural electrical stimulation timed to converge in the spinal cord promotes lasting increases in motor responses

**DOI:** 10.1113/JP274663

**Published:** 2017-08-20

**Authors:** Asht M. Mishra, Ajay Pal, Disha Gupta, Jason B. Carmel

**Affiliations:** ^1^ Burke‐Cornell Medical Research Institute 785 Mamaroneck Avenue White Plains New York 10605 USA; ^2^ Brain and Mind Research Institute and Departments of Neurology and Pediatrics Weill Cornell Medical College New York NY 10021 USA

**Keywords:** afferents, associative plasticity, corticospinal tract, electrical stimulation, motor cortex, spinal cord

## Abstract

**Key points:**

- Pairing motor cortex stimulation and spinal cord epidural stimulation produced large augmentation in motor cortex evoked potentials if they were timed to converge in the spinal cord.
- The modulation of cortical evoked potentials by spinal cord stimulation was largest when the spinal electrodes were placed over the dorsal root entry zone.
- Repeated pairing of motor cortex and spinal cord stimulation caused lasting increases in evoked potentials from both sites, but only if the time between the stimuli was optimal.
- Both immediate and lasting effects of paired stimulation are likely mediated by convergence of descending motor circuits and large diameter afferents onto common interneurons in the cervical spinal cord.

**Abstract:**

Convergent activity in neural circuits can generate changes at their intersection. The rules of paired electrical stimulation are best understood for protocols that stimulate input circuits and their targets. We took a different approach by targeting the interaction of descending motor pathways and large diameter afferents in the spinal cord. We hypothesized that pairing stimulation of motor cortex and cervical spinal cord would strengthen motor responses through their convergence. We placed epidural electrodes over motor cortex and the dorsal cervical spinal cord in rats; motor evoked potentials (MEPs) were measured from biceps. MEPs evoked from motor cortex were robustly augmented with spinal epidural stimulation delivered at an intensity below the threshold for provoking an MEP. Augmentation was critically dependent on the timing and position of spinal stimulation. When the spinal stimulation was timed to coincide with the descending volley from motor cortex stimulation, MEPs were more than doubled. We then tested the effect of repeated pairing of motor cortex and spinal stimulation. Repetitive pairing caused strong augmentation of cortical MEPs and spinal excitability that lasted up to an hour after just 5 min of pairing. Additional physiology experiments support the hypothesis that paired stimulation is mediated by convergence of descending motor circuits and large diameter afferents in the spinal cord. The large effect size of this protocol and the conservation of the circuits being manipulated between rats and humans makes it worth pursuing for recovery of sensorimotor function after injury to the central nervous system.

## Key points

- Pairing motor cortex stimulation and spinal cord epidural stimulation produced large augmentation in motor cortex evoked potentials if they were timed to converge in the spinal cord.
- The modulation of cortical evoked potentials by spinal cord stimulation was largest when the spinal electrodes were placed over the dorsal root entry zone.
- Repeated pairing of motor cortex and spinal cord stimulation caused lasting increases in evoked potentials from both sites, but only if the time between the stimuli was optimal.
- Both immediate and lasting effects of paired stimulation are likely mediated by convergence of descending motor circuits and large diameter afferents onto common interneurons in the cervical spinal cord.

**Abbreviations**
AUC: area under the curve
CST: corticospinal tract
DREZ: dorsal root entry zone
EEG: electroencephalogram
EMG: electromyogram
FDR: false discovery rate
i.p.: intraperitoneal
MEP: motor evoked potentials
MEP50: 50th percentile of the maximum for quantification
STDP: spike‐timing dependent plasticity
TMS: transcranial magnetic stimulation

## Introduction

When the nervous system is presented with paired sensory stimuli, it can associate them. This fundamental learning mechanism can also be leveraged to promote nervous system modulation through application of paired electrical stimulation. Modulation with paired stimulation can operate through control of an input and a target neuron, known as Hebbian (Hebb, 1949) or spike‐timing dependent plasticity (STDP; Feldman, 2012). In STDP protocols the timing of stimulation determines whether the synapse between two neurons becomes stronger (i.e. long term potentiation), or weaker (long‐term depression). Alternatively, multiple circuits can converge on a common target. The rules for such convergence are not as well understood, even though this is likely a mechanism for many protocols (Harel & Carmel, 2016).

The spinal cord is an attractive target for paired stimulation to promote motor function. The spinal cord has intrinsic circuitry that can perform complex movement independent of brain control (Miri *et al*. 2013). In addition, the spinal cord can learn and acquire new skills (Wolpaw, 2010). Finally, spinal cord stimulation can promote restoration of voluntary movement. In rodents (Courtine *et al*. 2009; Shah *et al*. 2016), monkeys (Capogrosso *et al*. 2016), and humans (Angeli *et al*. 2014) with injuries that partially spare brain to spinal cord connections, spinal cord epidural stimulation enabled movements that could not be achieved without stimulation. Importantly, rats regained most function if the spinal cord stimulation was provided at the time of locomotor training (Minev *et al*. 2015). Likewise, restored function in monkeys was strongest when spinal cord stimulation was given at the time leg motor cortex was activated (Capogrosso *et al*. 2016). These studies suggest a large capacity for sensorimotor skill encoded by the spinal cord that can be recruited by convergent motor and sensory activity.

We targeted the convergence of descending motor and local sensory circuits in the spinal cord. Previous attempts to alter spinal cord function through paired stimulation have largely used control of motor cortex and motoneurons to alter the strength of the synapse in between (Taylor & Martin, 2009; Bunday & Perez, 2012; Nishimura *et al*. 2013). The corticomotoneuronal system is an attractive target because it uses the better known rules of STDP by pairing input corticospinal neurons and their target motoneurons. However, only 15–20% of primate corticospinal projections target motoneurons; the rest project largely to neurons in the deep dorsal horn and intermediate zone of the spinal cord (Lemon & Griffiths, 2005).

We paired motor cortex stimulation to activate descending motor systems and spinal cord epidural stimulation, which selectively activates large diameter afferents (Rattay *et al*. 2000; Capogrosso *et al*. 2013). The termination of the corticospinal axons in the cervical spinal cord largely overlap with the large diameter afferents, which encode proprioception and muscle length and tension (Tan *et al*. 2012). Interaction of these two systems in the spinal cord is crucial for the execution of skilled movement (Arber, 2012; Takeoka *et al*. 2014). In addition, the corticospinal system and muscle afferents compete with one another in the spinal cord, further evidence for strong interactions between the systems (Jiang *et al*. 2016).

We hypothesized that cervical epidural electrical stimulation would augment cortical MEPs when paired to converge in the spinal cord. We observed two robust effects of this paired stimulation. First, spinal cord stimulation, given at an intensity below the threshold for evoking an MEP, augmented motor cortex evoked responses at the time it was delivered. This immediate effect was critically dependent on timing and the location in the spinal cord where the stimulation was applied. Second, motor cortex and spinal cord stimulation applied repeatedly over five minutes produced a robust augmentation of cortical and spinal MEPs lasting up to an hour. This lasting effect was observed only when the optimal timing was used. Thus, the repetitive stimulation induced an effect similar to associative learning that was likely mediated by interaction of the descending motor system and large diameter afferents at the level of the cervical spinal cord. These results add to our understanding of how paired stimulation affects motor circuits in the spinal cord and provide strong physiological evidence for a paired stimulation approach that can be applied for recovery of motor function after central nervous system injury.

## Methods

### Ethical approval

All experimental procedures were in full compliance with the approved Institutional Animal Care and Use Committee protocol of Weill Cornell Medicine. Adult female Sprague‐Dawley rats (28 female rats aged 99 ± 6 days (mean ± SEM) with an average weight of 275 ± 6 g; Charles River) were used. The animals were housed in individual cages with free access to food and water on a 12 h light–dark cycle^−1^.

### General surgical methods

Rats underwent two surgeries – implantation of electrodes in a survival surgery and a terminal physiology experiment. Anaesthesia was induced via intraperitoneal (i.p.) injection of a mixture of ketamine (90 mg kg^−1^) and xylazine (10 mg kg^−1^). This combination was used in order to preserve motor responses (Zandieh *et al*. 2003; Musizza *et al*. 2007; Englot *et al*. 2008). Carprofen (5 mg kg^−1^) was administered before and after the survival surgery to alleviate pain. Anaesthesia levels were monitored by respiration and heart rate and responses to foot pinch. For the physiology experiment, an i.p. (polyethylene PE 50 tubing, Instech, Plymouth Meeting, PA, USA) catheter was placed for continuous infusion of diluted ketamine in order to maintain stable levels of anaesthesia. Anaesthesia was maintained by a low dose (Zandieh *et al*. 2003) of 50–65 mg kg^−1^ h^−1^ continuous infusion of ketamine through the i.p. catheter. We choose this rate based on previous studies in which 50–65 mg kg^−1^ body weight of ketamine was given every hour (Englot *et al*. 2009; Carmel *et al*. 2010b). Animals were placed on water circulating heating pads (Gaymar Industries, Inc., Orchard Park, NY, USA) to maintain body a temperature of 37.5°C as measured continuously by a rectal probe (FHC Inc., Bowdoin, ME, USA).

### Motor cortex stimulation and recording of evoked responses

**Figure 1:**
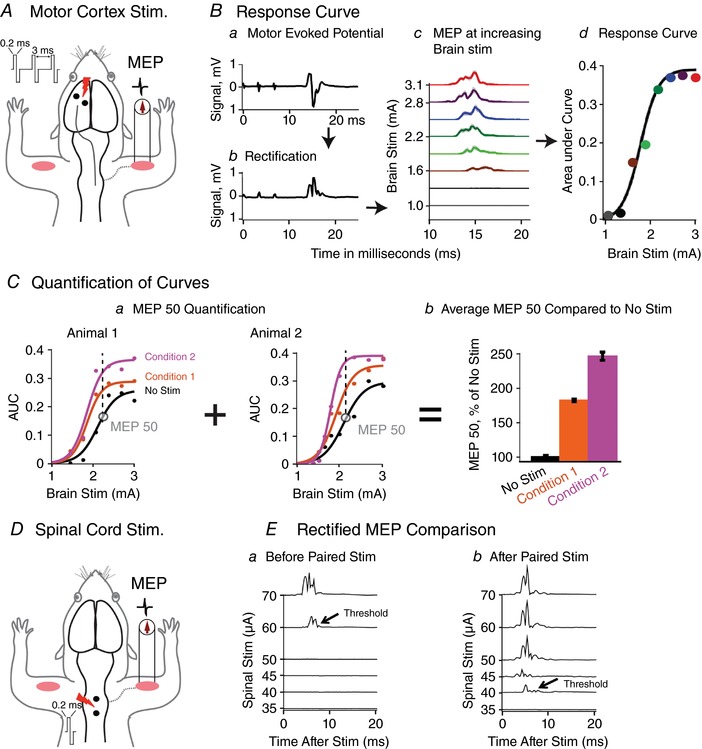
Stimulation, recording and quantification of MEPs *A*, motor cortex stimulation. Forelimb motor cortex was stimulated using epidural stainless steel screw electrodes, and MEPs were recorded from biceps brachii. A train of three biphasic pulses, each 0.2 ms long was delivered at 333 Hz. *B*, response curve. *Ba*, response to cortical stimulation shows small stimulation artifacts followed by MEP. *Bb*, MEP rectified. *Bc*, motor cortex is stimulated with increasing intensity from threshold to saturation (∼1 mA to 3 mA). Raw MEPs were rectified and averaged over 10 trials at each cortical stimulus intensity. *Bd*, the area under the curve of the rectified MEP (*y‐*axis) is plotted against the cortical stimulation intensity (*x‐*axis) to produce a characteristic ‘S’ shaped response curve, which is fitted using previously described methods. *C*, quantification of curves. *Ca*, curves acquired under the same experimental conditions in different animals were quantified using the 50th percentile of the baseline (no spinal stimulation) MEP. *Cb*, the MEP50 was averaged across animals and expressed as a percentage of the baseline (e.g. no spinal stimulation). Condition 1 = Spinal stimulation at 75% of the spinal threshold; Condition 2 = Spinal stimulation at 90% of the spinal threshold. *D*, spinal threshold. Two silver ball electrodes were placed on the dura overlying the dorsal cervical spinal cord and spinal MEPs recorded from biceps muscle. *E*, spinal threshold. Spinal stimulation intensity was adjusted to determine the threshold for provoking a short‐latency MEP in >50% of trials. *Ea*, spinal threshold before modulation. *Eb*, after modulation, the threshold was lower, indicating increased spinal excitability.

The sites of electrical stimulation and recording are shown in Fig. 1 *A*. One week before testing the effects of paired electrical stimulation, epidural cortical stimulating electrodes were placed. Rats were anaesthetized and head‐fixed in a stereotaxic frame (David Kopf Instruments, Tujunga, CA, USA). The skull was exposed and burr holes made using a Jobber Drill (number 60, Plastics One, Roanoke, VA, USA) without disturbing the dura mater. Stainless steel screw electrodes (1.19 mm diameter with a flat tip; Plastics One) were implanted over the forelimb area of motor cortex in one hemisphere at two locations: 1.0 mm anterior, and 2.0 mm lateral; and 3.0 mm anterior and 4.0 mm lateral to bregma), as determined by a previous mapping study (Brus‐Ramer *et al*. 2009). The screw electrodes were attached in advance to a head connector (Plastics One) that was secured with skull screws and dental acrylic.

To assay the descending motor systems, we stimulated motor cortex and measured MEPs from contralateral biceps muscle. For motor cortex stimulation, a train of three biphasic square wave pulses was used (each pulse of 0.2 ms for each polarity; interstimulus interval of 3 ms; Fig. 1 *A*); an Isolated Pulse Stimulator (A‐M Systems, Model 2100, Sequim, WA, USA) was used. Three pulses were used, because a single pulse causes activation of both motor cortex and subcortical structures (Patton & Amassian, 1954; Ra *et al*. 1988). Therefore, a pulse train was needed in order for temporal summation to selectively activate motor cortex (Taniguchi *et al*. 1993). We kept the train short, three biphasic pulses, as in a previous study (Brus‐Ramer *et al*. 2007), to facilitate determination of the best timing of paired brain and spinal cord stimulation. In addition, we also compared latencies with three pulses to single pulses delivered over motor cortex. For testing, trains of stimuli were delivered every 2 s to allow recovery of responses (Carmel *et al*. 2010a).

### MEP quantification

To measure cortical MEPs, we inserted electrodes into the biceps muscle bilaterally to record an electromyogram (EMG), as in previous studies (Brus‐Ramer *et al*. 2009). Supple stainless steel braided wire (Cooner Wire, catalogue number AS 634, Chatsworth, CA, USA) was deinsulated for 1 mm and threaded through the muscle using a 22.5‐gauge needle. To verify proper muscle electrode placement, the elbow was extended to evoke a stretch reflex‐evoked increase in EMG activity. Electrodes were knotted on either side to keep the deinsulated portion in the centre of the muscle.

EMG was continuously acquired with a differential AC amplifier system (A‐M Systems, Model 1700), amplified at a gain of 1000 and bandpass filtered between 1 and 1000 Hz which has been used for EMG recording (Basmajian & Deluca, 1985). A data acquisition system (CED Micro 1401, Cambridge Electronic Design Ltd, Cambridge, UK) running recording software (Signal 5.08, CED Ltd) was used to record at a sampling rate of 5000 Hz. It was also used to record the precise stimulation onset time points and synchronize it with the EMG data. MEPs were extracted, processed and quantified using a customized MATLAB (MathWorks, Natick, MA, USA) software. Raw EMG signals (Fig. 1 *Ba*) were rectified (Fig. 1 *Bb*) and then averaged across 10 trials at each stimulus intensity. We used the first 25 ms of EMG data after stimulation for quantification; the EMG response diminished to baseline within this time period. The area under the curve (AUC) was calculated for averaged MEPs (Fig. 1 *Bc* and *d*). Recordings were taken at regular intervals, from a low cortical stimulus intensity that does not produce any motor response (subthreshold; ∼0.5 mA), to high intensity (∼3.0 mA) that saturates the MEP. Plotting AUC on the *y*‐axis and cortical stimulus intensity on *x*‐axis produced a characteristic sigmoidal response curve (Fig. 1 *Bd*).

Cortical MEPs were quantified using these response curves. We used the MEP AUC value at the 50th percentile of the maximum for quantification (MEP50 in Fig. 1 *C*) (Song *et al*. 2016). This method has been used for assessing cortical excitability (Nardone *et al*. 2015) as well as spinal cord excitability (Pierrot‐Deseilligny & Burke, 2012). The MEP50 value of the baseline curve (e.g. no spinal stimulation) determined how the response curves were compared (Fig. 1 *Ca*); the MEP50 values at the same cortical stimulus intensity were compared across conditions for each rat. The MEP50 values from different rats were then averaged and expressed as a percentage of the baseline (no spinal stimulation; Fig. 1 *Cb*). For statistical comparisons, the raw MEP50 values (not expressed as a percentage of baseline) were used.

We also measured the stimulus intensity threshold for evoking a spinal MEP. After spinal electrode placement on the midline over C5–C6 (see next section), we applied a single biphasic pulse of spinal cord stimulation to determine the amount of current needed to evoke a spinal MEP in 50% of trials (Fig. 1 *D* and *E*). This stimulus intensity was defined as the spinal cord stimulation threshold.

### Cervical spinal cord stimulation

The cervical spinal cord was stimulated epidurally with two custom silver ball electrodes (0.75 mm in diameter) using an A‐M Systems, Model 2100 stimulator. The anaesthetized rat was head‐fixed in a stereotactic frame (Kopf), and the T1 spinous process was exposed and clamped. The spinal cord was exposed with laminectomies from C3 through C7. Mineral oil was used to fill the space over the spinal cord to protect it and to keep the electrodes electrically isolated from one another. The stimulating electrodes were placed at various rostrocaudal positions over the cervical spinal cord. In addition, the mediolateral position was also tested in midline, over the dorsal root entry zone (DREZ), and over the C5 and C6 dorsal roots.

### Overview of physiology experiments

We performed two types of paired stimulation experiments: immediate effects at the time of paired stimulation and lasting effects of repetitive pairing. For the immediate effects, multiple parameters were tested in each rat (*n* = 16), such as latency, intensity, and electrode position. For the lasting effects, the optimal latency of pairing from initial experiments were tested, and then only one repetitive pairing experiment was performed in each rat (*n* = 12). Rats were randomized to experimental and control conditions. We also performed recordings of brain, spinal cord and muscle in response to brain and spinal cord stimulation in order to measure latency.

### Immediate effects of paired motor cortex and cervical spinal cord stimulation

In the first group of experiments, we measured the immediate effects of subthreshold cervical spinal cord stimulation on cortical MEPs. The MEP curves of motor cortex stimulation alone (no spinal stimulation) were compared against the responses to paired motor cortex and spinal cord stimulation. We measured the effects of several variables in paired stimulation, including (a) latency, (b) electrode position, and (c) polarity.

**Figure 2:**
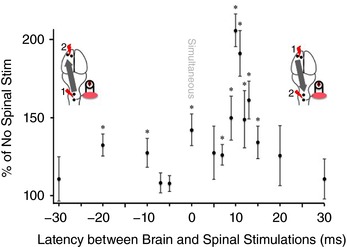
Effects of paired stimulation depend on timing We tested different latencies between motor cortex and spinal cord stimulation from −30 ms to 30 ms. As indicated by the insets, negative times indicate that spinal cord was stimulated before motor cortex stimulation and positive times indicate that spinal cord was stimulated after motor cortex stimulation. Spinal cord stimulation was set at 90% of threshold. MEPs from paired stimulation were compared with brain stimulation only using the methods shown in Fig. 1. Maximum augmentation was found when spinal cord stimulation was delivered 10 ms after motor cortex stimulation. MEPs were significantly elevated at the time points indicated, as measured by multiple paired *t* test compared to no spinal stimulation baseline with correction for multiple comparisons using FDR (*n* = 24, as detailed in Methods). [Color figure can be viewed at wileyonlinelibrary.com]

(a) For latency, we stimulated the spinal cord at various time intervals from 30 ms before brain stimulation to 30 ms after brain stimulation (Fig. 2). The number of animals tested at each latency is shown here as latency in milliseconds (number of animals): −30 (2), −20 (17), −10 (18), −7 (4), −5 (5), 0 (17), 5 (4), 7 (14), 9 (9), 10 (19), 11 (14), 12 (7), 13 (16), 15 (8), 20 (11), 30 (7). (b) For location, we varied the rostrocaudal location and the mediolateral location of the spinal stimulating electrodes. In the mediolateral position, we stimulated over the midline, over each dorsal root entry zone, and over the C5 and C6 dorsal roots (*n* = 3 each). (c) For polarity, we used one electrode over the dorsal spinal cord and a hook electrode in the skin of the abdomen (*n* = 2). The spinal cord was stimulated with biphasic (as described above), cathodal, and anodal, 0.2 ms square wave pulses. Since biphasic stimulation produced similar augmentation compared to cathodal stimulation (data not shown), we have used biphasic stimulation throughout. This is based on the logic that biphasic waveforms produce smaller electrode changes compared with uniphasic stimulation (Walcott *et al*. 1995).

### Lasting effects of repetitive paired brain and spinal cord stimulation

**Figure 3:**
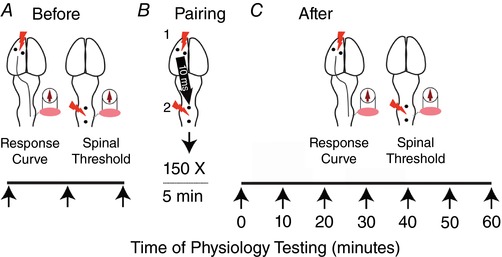
Repetitive paired motor cortex and spinal cord stimulation protocol *A*, physiology testing includes motor cortex evoked response curves and spinal cord stimulation current needed to evoke MEP (spinal threshold). Physiology testing was performed before and after paired stimulation. *B*, motor cortex is stimulated just above the threshold for evoking MEP and the spinal cord is stimulated 10 ms later at 90% of threshold for evoking MEP. This pairing was performed for 5 min, a total of 150 paired stimulations. *C*, after stopping pairing, physiology testing was performed immediately after and at 10 min intervals thereafter. [Color figure can be viewed at wileyonlinelibrary.com]

For the second set of experiments, we performed repeated pairing of motor cortex and spinal cord stimulation, as shown in Fig. 3. There are two critical differences between this modulation and the immediate effects described above. First, the pairing of brain and spinal cord stimulation was repeated 150–300 times. Second, the physiological measures of brain and spinal cord excitability that were collected at baseline and again after the paired stimulation, were performed by stimulating the brain only and then the spinal cord only (i.e. this was single site stimulation; no paired stimulation for these two outcome measures). At baseline and again after the repetitive pairing, cortical MEPs and spinal thresholds were measured singly and independently (Fig. 3). Before modulation, we created a response curve using motor cortex stimulation only (pre‐pairing baseline). We also measured the spinal threshold for evoking an MEP (Figs 1 *Ea* and 3 *A*). We then performed repeated pairing of motor cortex and spinal cord stimulation (Fig. 3 *B*). Motor cortex stimulation was performed at the threshold for evoking a cortical MEP followed 10 ms later by spinal cord stimulation performed at 90% of the threshold for generating a spinal MEP. After modulation we again created a response curve using motor cortex stimulation only and separately measured spinal threshold (Figs 1 *Eb* and 3 *C*). Each of these measures were taken immediately after pairing and every 10 min thereafter up to 60 min. In control experiments, we tested, brain or spinal cord stimulation alone during the 5 min of repetitive stimulation.

We assessed the effect of three parameters for the repetitive pairing protocol (Fig. 7). (a) Duration of pairing: we paired midline spinal cord stimulation 10 ms after cortical stimulation for either 5 (Fig. 7 *A*) or 10 min (Fig. 7 *B*), for a total of 150 or 300 paired stimuli respectively. (b) Position of spinal stimulation: midline *vs*. dorsal root entry zone (Fig. 7 *C*) for 5 min (150 paired stimuli) of paired stimulation. (c) Latency between the cortical and spinal stimulation: in a control experiment, we performed the 5 min midline protocol (Fig. 7 *A*) but with 100 ms between the cortical and spinal cord stimulation during pairing (a time that did not cause any immediate effects).

### Experiments to test mechanisms of paired stimulation

**Figure 4:**
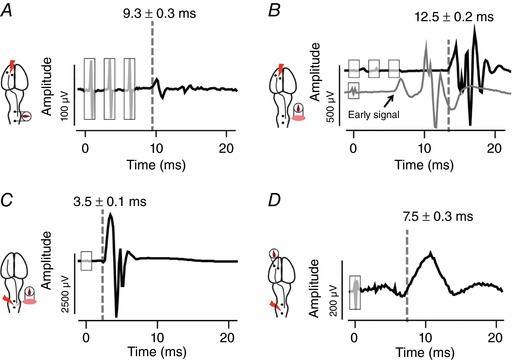
Latency of responses to motor cortex and dorsal spinal cord epidural stimulation The latencies are averages across five rats; the responses are a single representative trial. *A*, cervical spinal cord dorsum cord potential after motor cortex stimulation. *B*, MEP from contralateral biceps after motor cortex stimulation with three pulses (black trace) and single pulse (grey trace). Single pulse stimulation at motor cortex evokes an early as well as a late response. *C*, MEP from biceps after suprathreshold, single pulse stimulation delivered at the midline of the cervical spinal cord. *D*, cortical potential (EEG) to middle spinal cord stimulation. Spinal cord stimulation and cord dorsum potentials were recorded through ball electrodes. Motor cortex stimulation and EEG recordings were performed through stainless steel screw electrodes implanted over the motor cortex. Shaded rectangles indicate stimulation artifacts. [Color figure can be viewed at wileyonlinelibrary.com]

We tested latencies of responses to brain or spinal cord stimulation in order to investigate how they might interact during pairing. The methods and latencies are shown in Fig. 4. Figure 4 *A* shows recording of the cervical spinal cord dorsum potential after motor cortex stimulation. Motor cortex was stimulated as described above at the threshold for evoking an MEP using implanted screw electrodes, and spinal cord dorsum potentials were recorded over the midline of C5 and C6 (A‐M Systems, Model 1700) using the same silver ball electrodes used for stimulation (Schaible *et al*. 1986). Figure 4 *B* shows the latency for cortical MEPs. Cortical stimulation at threshold was used to provoke an MEP in biceps. The latency is shown from the onset of the three pulse train (Fig. 1 *A*). Figure 4 *C* shows the latency of spinal MEPs. As described above for spinal threshold, the spinal cord was stimulated in the midline of C5 and C6, and an MEP recorded in biceps. Finally, in Fig. 4 *D*, cortical potentials (EEG) were recorded, in differential mode (Mishra *et al*. 2011), after cervical spinal cord stimulation. The spinal cord was stimulated at the spinal threshold using the ball electrodes over the midline of C5 and C6, and cortical potentials were measured using screw electrodes (same electrodes that were used for cortex stimulation).

To determine if the stimulation of the spinal cord recruits afferents, we performed two additional experiments. First, we recorded biceps MEPs after stimulation at C5–C6 in the midline of the spinal cord, DREZ, and dorsal roots. Spinal MEPs were generated using a single biphasic pulse at the threshold current intensity. Second, we performed repeated stimulation at each of these sites with 15 pulses at 100 Hz. This was done to assess response to repetitive stimulation, which causes depression of MEPs if afferents are stimulated (Sharpe & Jackson, 2014). At the conclusion of experiments, rats were killed with an overdose of pentobarbital (150 mg kg^−1^; Euthasol, Virbac AH Inc., Fort Worth, TX, USA).

### Statistical analysis

All analysis was performed with SPSS (Version 22). We first tested distribution of the data (normality) using the Shapiro‐Wilk test. All the data for latency, electrode position, and lasting effects had normal distribution; therefore, we used parametric statistics. For tests of latency, each timing was compared to its own baseline recruitment curve obtained without stimulation. Since separate experiments were performed for each latency, these were independent Student's paired *t* tests. For mediolateral position, each position was compared against each other with ANOVA with correction for multiple comparisons. For the repeated pairing protocol, time points were compared to the no‐stimulation baseline with paired *t* tests corrected for multiple comparisons. Correction for multiple comparisons was performed using the Benjamini‐Hochberg false discovery rate (FDR; McDonald, 2014) (with 0.2 FDR). Significance was set at *P* < 0.05.

## Results

### Effect of latency between brain and spinal cord stimulation

We hypothesized that subthreshold spinal epidural stimulation would augment cortical MEPs when the two were timed to converge in the spinal cord. In these experiments, the spinal cord was always stimulated in the midline at the C5–C6 spinal level with an intensity that was 90% of the spinal threshold for provoking a biceps MEP. We tested various pairing latencies to determine the optimal latency for pairing, as shown in Fig. 2. As indicated by the inset schematics, negative times indicate spinal cord stimulation before motor cortex stimulation and positive times indicate spinal cord stimulation after motor cortex stimulation.

A single biphasic pulse of subthreshold spinal epidural stimulation significantly augmented the biceps MEP produced by suprathreshold motor cortex stimulation (Fig. 2). MEPs were significantly increased at −20 ms (*P* = 0.001) and −10 ms (*P* = 0.026), when the spinal cord was stimulated first and motor cortex second, as well as simultaneous (*P* = 0.002) motor cortex and spinal cord stimulation and when the cervical spinal cord was stimulated 7, 9, 10, 11, 12, 13 and 15 ms after motor cortex (*P* values = 0.007, 0.013, 1.01 × 10^−10^, 5.3 × 10^−5^, 0.021, 3.2 × 10^−4^, and 0.026, respectively). There was no significant augmentation of cortical MEPs when spinal stimulation was given 30 ms before or after brain stimulation or when the spinal cord was stimulated 5 ms or 7 ms before motor cortex. The augmenting effect of spinal cord stimulation was particularly prominent at 10 ms; MEPs were increased by an average of 205 ± 11% in the 19 rats that were tested at this latency. No depression of cortical MEPs by spinal cord stimulation was observed at any of the latencies tested.

To understand why the modulating effect of subthreshold spinal cord stimulation peaked at 10 ms after brain stimulation, we measured latencies of responses to each site of stimulation. The latencies shown in Fig. 4 are the averages of 10 responses each from four animals, while the waveforms are for a representative trial in one animal. As shown in Fig. 4 *A*, we used ball electrodes to record C5–C6 spinal cord responses (cord dorsum potential) to cortical stimulation. The latency of the response was 9.3 ± 0.3 ms after the onset of the train of three pulses of cortical stimulation. This latency was strikingly similar to the 10 ms latency that was optimal for spinal stimulation to augment cortical MEPs.

We measured MEP latency after motor cortex stimulation delivered at threshold intensity with one or three biphasic pulses. The MEP latency after motor cortex stimulation with three pulses was 12.5 ± 0.2 ms (Fig. 4 *B*, black trace). After motor cortex stimulation with a single biphasic pulse, there were two peaks in the MEP, a smaller early response at 5.8 ± 0.1 ms and a later larger MEP latency after motor cortex stimulation with single pulse was 10 ± 0.2 ms (Fig. 4 *B*, grey trace). Also single pulse motor cortex stimulation threshold was 2.2 times higher than motor cortex stimulation with three pulses (1.8 ± 0.2 mA). As single pulse stimulation produces an early response that could be mediated by activation of the brainstem (see Methods), we used three pulses over motor cortex for all paired stimulation experiments. This limits ascertainment of latency, since one does not know which of the three pulses triggered the volley, but it makes it more likely that the MEP is a cortical (as opposed to brainstem) response. We also recorded MEPs after spinal cord stimulation, which we call spinal MEPs. As previously stated, all neuromodulation is performed with spinal cord stimulation set at an intensity below the threshold for evoking a spinal MEP. If the spinal cord is stimulated at a higher intensity, however, a spinal MEP is evoked. Using a single biphasic pulse at the spinal threshold, we recorded an MEP 3.5 ± 0.1 ms after the onset of spinal cord stimulation (Fig. 4 *C*). Cortical potentials (EEG) recorded after threshold spinal epidural stimulation had a latency of 7.5 ± 0.3 ms (Fig. 4 *D*). Interactions in motor cortex should have latencies much longer than 10 ms because the time for cortical potentials plus the time for cortical MEPs is on the order of 20 ms. These data support our hypothesis that spinal cord stimulation augments motor cortex MEPs via convergence of afferent and descending motor pathways in the spinal cord, and not in cortex.

### Position of spinal cord stimulation electrodes

**Figure 5:**
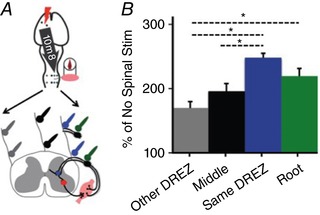
Paired stimulation depends on mediolateral position of spinal stimulating electrodes *A*, two ball electrodes were placed in rostrocaudal orientation over the C5–C6 segment of the spinal cord. Spinal cord stimulation was set at 90% of the lowest threshold. *B*, stimulation of the spinal cord DREZ on the same side as the MEP measurement (Same DREZ, blue) produces the largest increase in MEPs compared to baseline (no spinal cord stimulation). This was followed by the response when stimulation was performed over the dorsal root (green), stimulation over the middle (black), and the DREZ opposite the side from which the biceps was recorded (grey). All values were significantly increased from baseline and also from each other (ANOVA with *post hoc* correction for multiple comparisons, *F* = 37.22, *P* = 2.5 × 10^−7^, *n* = 4 animals).

If spinal cord epidural stimulation acts via excitation of afferents, this creates predictions about where on the spinal cord it will be most effective to stimulate. Stimulation over the DREZ (Fig. 5, blue) on the side of the biceps being measured is predicted to most strongly augment cortical MEPs because it places the electrodes closest to the site of convergence with CST and other crossed motor connections. Placing the stimulating electrodes over the dorsal roots themselves (Fig. 5, green) tests whether modulation is mediated through afferents. The electrodes are placed off of the spinal cord, which is bathed in mineral oil, insulating against flow of current except through the C5 and C6 dorsal roots. We also tested stimulation in the midline (Fig. 5, black) and at the DREZ on the side of the spinal cord away from the stimulating electrode (Fig. 5, grey). Spinal cord stimulation intensity was held constant (90% of threshold at DREZ) at each of these sites and followed cortical stimulation by 10 ms. As shown in Fig. 5 *B*, stimulation of the DREZ close to the MEP measurement led to maximum augmentation of the cortical MEPs compared to no spinal cord stimulation baseline (248 ± 7%; blue), followed by dorsal root (219 ± 12%; green), midline (196 ± 12%; black), and the DREZ opposite side to the MEP recording (172 ± 10%; grey). There was a strong difference among the groups (ANOVA with *post hoc* correction for multiple comparisons, *F* = 37.22, *P* = 2.5 × 10^−7^); the individual comparisons that were significant after *post hoc* correction are shown in Fig. 5 *B*).

**Figure 6:**
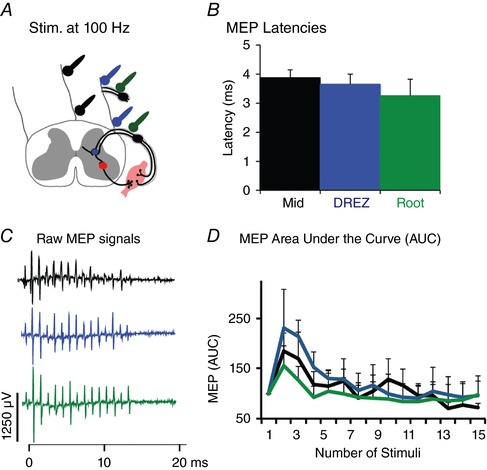
Similar latency and modulation to high frequency stimulation trains across three different sites over spinal cord *A*, like Fig. 5, ball electrodes were placed at midline (black), DREZ (blue), or on the dorsal root (green), Spinal cord stimulation was delivered at and MEPs were recorded from biceps. *B*, latency of MEPs at each site of stimulation after a single biphasic pulse delivered at threshold are not different from one another (ANOVA with repeated measure, *F* = 1.42, *P* = 0.27, *n* = 4 animals). *C*, representative MEPs evoked by trains of 15 biphasic stimuli delivered at 100% of the spinal threshold intensity at 100 Hz. *D*, area under the curve of MEPs after stimulation at above‐mentioned three places over the spinal cord. Facilitation was observed at the second pulse and suppression on subsequent responses. Similar responses to trains were observed from the three sites of stimulation (ANOVA with repeated measure, *F* = 0.26, *P* = 0.76; *n* = 4).

To understand the mechanism by which spinal cord stimulation augments motor cortex responses, we tested the latencies of spinal MEPs from suprathreshold spinal cord stimulation at the three sites indicated in Fig. 6 *A*. If spinal cord stimulation at each of these sites acts by activation of large diameter afferent fibres, as suggested by modelling studies (Capogrosso *et al*. 2013), the latencies between each of the stimulation sites should be similar. Also, if MEPs are triggered by afferent stimulation, then the latencies should be similar to the latencies of H‐reflex; which is ∼4 ms for the extensor carpi radialis after deep radial nerve stimulation in rats (Hosoido *et al*. 2009; Tan *et al*. 2012). Indeed, as shown in Fig. 6 *B*, the latencies to onset of spinal MEPs were not different between stimulation in the middle of the spinal cord (3.87 ± 0.26 ms; black), DREZ (3.64 ± 0.35 ms; blue), and dorsal roots (3.25 ± 0.55 ms; green; ANOVA with repeated measure, *F* = 1.42, *P* = 0.27, *n* = 4 animals) and are similar to the latency of a forelimb H‐reflex in rats (Tan *et al*. 2012). These results suggest that the same circuits were activated by stimulation at each of these sites, including stimulation of the dorsal roots only.

To further examine if the MEP might be mediated by activation of afferents, we used a stimulation protocol that produces a characteristic response pattern for afferents (Sharpe & Jackson, 2014). A high frequency (100 Hz) and suprathreshold stimulation of afferents causes an increase of responses from the first to the second pulse and depression of MEPs in subsequent pulses (*n* = 15 pulses). This characterizes responses to high frequency stimulation and contrasts with motor neuron stimulation, which causes either continued augmentation with subsequent or at least lack of depression (Jackson *et al*. 2006; Sharpe & Jackson, 2014). We stimulated the spinal cord with a biphasic and suprathreshold pulse and in the locations indicated by Fig. 6 *A*. Example spinal MEPs in right biceps brachii to repetitive spinal cord stimulation shows augmentation with the second pulse and then a gradual decrease (Fig. 6 *C*). Quantification of MEPs using area under the curve of across four animals is shown in Fig. 6 *D*. The MEPs did not differ between stimulation of the midline, DREZ, or dorsal roots (ANOVA with repeated measures, *F* = 0.26, *P* = 0.76). This further supports recruitment of large diameter afferents as a likely mechanism for augmentation of cortical MEPs by spinal cord stimulation. In addition, the response to repetitive stimulation was similar at each of the sites, which corroborates the similarity in modulation of cortical MEPs (Fig. 5 *B*) and similar latencies of spinal MEPs (Fig. 6 *B*).

### Comparison with spinal stimulation parameters in current practice

Spinal cord stimulation is used clinically for pain management and experimentally for restoration of motor function. Several experimental protocols use continuous (tonic) epidural spinal cord stimulation in rodents (Minev *et al*. 2015) and patients with SCI (Harkema *et al*. 2011; Angeli *et al*. 2014). We compared our single pulse protocol with continuous stimulation at 40 Hz. Both protocols were performed at 90% of the spinal threshold for single pulse stimulation. The 40 Hz stimulation protocol gives pulses in 25 ms intervals, including cortical and spinal stimulation at the same time, and spinal stimulation 25 ms before and after cortical stimulation. While 40 Hz spinal cord stimulation increased the cortical MEPs (213 ± 40% of no stimulation), the effects were slightly larger with a single pulse spinal stimulation delivered at the optimal latency of 10 ms (220 ± 47%; paired *t* test, *P* = 0.03, *n* = 4 animals). Thus, a single pulse of spinal cord stimulation delivered at the optimal latency caused a slightly larger increase in MEPs than continuous stimulation.

Finally, we tested the effects of modulating the intensity of spinal cord stimulation. Clinical spinal cord stimulators often use stimulation intensity well below the motor threshold. Like the other modulation described thus far, we tested an intensity of 90% of motor threshold as well as 75% of motor threshold. We found stronger augmentation with spinal stimulation at 90% of threshold current (191 ± 15%) *versus* stimulation at 75% of the threshold current (163 ± 9%; paired *t* test, *P* = 0.03, *n* = 4 animals). Thus, intensity is another important variable for effective pairing of motor cortex and spinal cord stimulation.

### Lasting effects of paired stimulation

Thus far, we have demonstrated that augmentation of cortical MEPs occurs at the time that the spinal cord is stimulated. We tested the hypothesis that repeatedly pairing motor cortex and spinal cord stimulation would cause lasting changes in cortical and spinal cord excitability. As shown in Fig. 3, we tested this hypothesis by measuring cortical MEPs and spinal thresholds at baseline (before modulation with pairing). We then paired motor cortex and spinal cord stimulation 10 ms later, every 2 s, for at least 5 min (150 pairs). Finally, we tested cortical MEPs and spinal thresholds directly after 5 min of pairing, and every 10 min thereafter until the responses returned to baseline (40–60 min).

**Figure 7:**
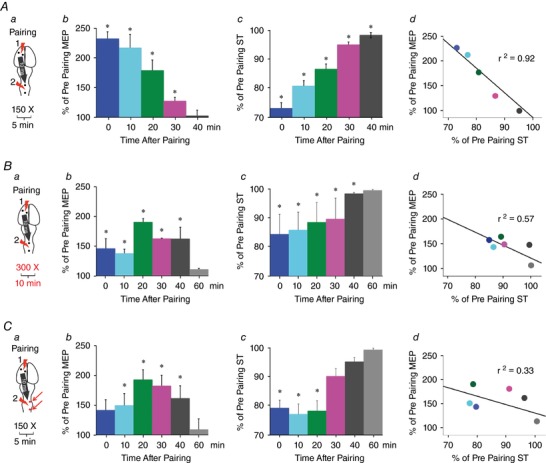
Repetitive pairing of motor cortex and spinal cord stimulation produces lasting increases in MEP and decreases in spinal threshold The experimental protocol shown in Fig. 3 was applied as shown in *A*. In the experiment shown in *B* the pairing time was increased to 10 min. In the experiment shown in *C* the spinal cord was stimulated at the DREZ. In each case the spinal cord was stimulated with a single biphasic pulse delivered at 90% of threshold and 10 ms after motor cortex stimulation delivered at motor threshold. *Aa*, paired stimulation protocol. *Ab*, large increases in MEPs immediately after stopping pairing (225 ± 23%), gradually diminished to baseline after 40 min. *Ac*, significant decreases in spinal thresholds after pairing (26 ± 2%, *t* test, *P* = 0.01) gradually diminished but were still significantly decreased at 40 min. *Ad*, there is a strong correlation between increase in motor evoked response and decrease in spinal thresholds (Pearson's correlation, *r* ^2^ = 0.92, *P* = 0.003). *Ba*, stimulation protocol. *Bb*, cortical MEPs were increased immediately after stopping pairing and peaked at 20 min (190 ± 5%) before diminishing to baseline at 60 min. *Bc*, significant decreases in spinal threshold immediately after pairing (15 ± 2%, *t* test, *P* = 0.05) returned to baseline at 60 min. *Bd*, correlation between increase in cortical MEP and significant decrease in spinal thresholds (Pearson's correlation, *r* ^2^ = 0.57, *P* = 0.05). *Ca*, paired stimulation protocol. *Cb*, increases in motor evoked responses immediately after stopping pairing peaked at 20 min (193 ± 16%) and gradually diminished to baseline after 60 min. *Cc*, spinal thresholds were significantly decreased (22 ± 1%, *t* test, *P* = 0.05) out to 20 min. *Cd*, correlation between increase in cortical MEP and decrease in spinal thresholds was not significant (Pearson's correlation, *r* ^2^ = 0.33, *P* = 0.172).

As shown in Fig. 7 *A*, repetitive pairing caused robust augmentation of cortical MEPs and decrease in spinal threshold. The size of cortical MEPs were strongly and significantly increased immediately after the 5 min of pairing (Fig. 7 *Ab*; 225 ± 23% of pre‐pairing baseline, *n* = 4 rats). Cortical MEPs were significantly elevated up to 30 min after the pairing had ended (*t* test for each time point values compared with baseline, corrected for multiple comparisons) and returned to baseline at 40 min. The increase in cortical MEPs was accompanied by a decrease in spinal threshold, indicating increased excitability immediately after 5 min of pairing, the spinal cord threshold was decreased by 26 ± 2% (Fig. 7 *Ac*; *t* test, *P* = 0.01) compared to baseline spinal threshold. The spinal cord threshold gradually increased to the end of testing at 40 min. If the increases in cortical MEPs were due to changes in spinal excitability, then there should be a strong correlation of the increase in cortical MEPs and the decrease in spinal threshold. This relationship was very strong and significant (Fig. 7 *Ad*; Pearson's correlation, *r* ^2^ = 0.92, *P* = 0.003).

We tested whether a longer duration of pairing would alter the strength or the duration of the effects. As shown in Fig. 7 *Ba*, the stimulation was increased to 10 min and 300 pairs of motor cortex and cervical spinal cord stimulation. After 10 min of pairing, there was an increase in cortical MEP, which was smaller in magnitude compared to the initial effects after 5 min of pairing. Maximum changes were observed at 20 min after stopping the pairing (Fig. 7 *Bb*; 190 ± 5% of pre‐pairing baseline; *n* = 4 rats) and stayed significantly elevated at 40 min. The cortical MEPs returned to baseline at 60 min. Spinal threshold show a significant decrease of 15 ± 2% (Fig. 7 *Bc*; *t* test, *P* = 0.05) below the baseline immediately after pairing stopped. The threshold increased slowly over the 40 min, returning to baseline at 60 min. Also, the correlation between the increase in motor responses and the decrease in spinal threshold was just above significance (Fig. 7 *Bd*; Pearson's correlation, *r* ^2^ = 0.57, *P* = 0.05).

Finally, we tested whether stimulation over the DREZ might alter the strength and duration of lasting effects. Since stimulation over the DREZ caused larger changes at the time of stimulation compared to midline stimulation (e.g. Fig. 5 *B*), we hypothesized that there would be larger and more durable effects of repetitive DREZ stimulation compared to midline. As shown in Fig. 7 *Ca*, paired stimulation was performed in the same manner as Fig. 7 *A* except the spinal stimulation electrodes were placed over the DREZ. This protocol did not significantly increase cortical MEPs immediately after pairing. Instead, maximum changes were observed 20 min after stopping pairing (Fig. 7 *Cb*; 193 ± 16% of pre pairing, *n* = 4 rats) and stayed significantly elevated up to 40 min and returned to baseline at 60 min. Unlike cortical MEPs, spinal threshold was most strongly affected at the end of the paired stimulation interval. The spinal threshold decreased to 22 ± 1% (*t* test, *P* = 0.05) of baseline immediately after pairing stopped and increased slowly, returning to baseline at 60 min (Fig. 7 *Cc*). There was no significant correlation between the increase in motor responses and the decrease in spinal threshold (Pearson's correlation, *r* ^2^ = 0.33, *P* = 0.17, Fig. 7 *Cd*).

### Control experiments

To determine whether the effects of motor cortex and spinal cord stimulation were due to their pairing at an effective latency, we tested paired stimulation using the same protocol as shown in Figs 3 and 7 *Aa* but at a latency of 100 ms instead of 10 ms (*n* = 4). In keeping with the data shown in Fig. 2, pairing at 100 ms had no immediate effect on cortical MEPs at the time that stimulation was performed (101 ± 9%; *n* = 4). When this pairing was done repeatedly over 5 min, there were no changes in cortical MEPs (102 ± 12%; *n* = 4) or in spinal thresholds (100 ± 1%; *n* = 4) immediately after stopping pairing. Cortical MEPS and spinal threshold values did not change over the next 30 min (data not shown). We also tested the effects of spinal cord stimulation alone, delivered at 90% of threshold, every 2 s over 5 min (150 single pulse spinal cord stimuli; *n* = 4). There were no immediate effects of spinal cord stimulation alone on cortical MEPs (99 ± 4%; *n* = 4). Similarly, we tested the effects of motor cortex stimulation alone over 5 min. There was no change in cortical MEPs (100 ± 7%; *n* = 4), and these values did not change over the following 30 min (data not shown). Thus, it is the pairing of motor cortex and spinal cord stimulation, rather than the separate effects of each, that is critical to the lasting effects. Effective pairing also critically relies on the proper latency between brain and spinal cord stimulation.

## Discussion

Pairing spinal cord stimulation after motor cortex stimulation creates immediate as well as lasting effects on cortical and spinal excitability. These results can be interpreted through our current working model – paired stimulation strengthens connections in the cervical spinal cord through interactions of descending motor pathways and sensory afferents. Here we discuss the evidence for the model, compare it to other paired stimulation protocols directed at the spinal cord, and suggest future applications.

### Convergence of descending efferents and spinal afferents

The time between motor cortex and spinal cord stimulation is critical for the immediate effects of paired stimulation, with 10 ms latency producing maximum augmentation (Fig. 2). There is striking concordance between the timing of optimal pairing (10 ms, Fig. 2) and the latency of the spinal cord dorsum potential recorded after motor cortex stimulation (9.3 ms ± 0.3 ms, Fig. 4 *A*). This is closely matched with the latency of the excitatory postsynaptic potentials between motor cortex and motoneurons in rats, averaged at 8.45 ms (Babalian *et al*. 1993). The effects at 10 ms were large (206%), and the augmenting effects of paired stimulation diminished quickly when the timing was altered to slightly before (e.g. 5 ms; 124%) or after (e.g. 15 ms; 131%) this peak. Thus, small alterations in pairing timing had large effects on the efficacy of paired stimulation. The similar timing of the descending volley and the peak pairing effect suggest convergence of motor cortex and large diameter afferent stimulation by spinal cord stimulation in the cervical spinal cord.

Three pieces of experimental evidence support the mathematical model (Capogrosso *et al*. 2013) and human spinal cord stimulation findings (Rattay *et al*. 2000) that spinal epidural stimulation recruits large diameter afferents. First, the maximal effect of pairing was achieved when the stimulating electrodes were placed over the DREZ close to the side of MEP measurement (Fig. 5 *B*). Second, suprathreshold responses are similar between DREZ, dorsal root and midline stimulation (Fig. 6 *B*). This suggests that stimulation on the dorsum of the spinal cord produces effects similar to stimulation of the dorsal roots themselves. Finally, rapid and repetitive stimulation at each of these sites produced a decrement of response (Fig. 6 *C* and *D*), a signature of afferent stimulation.

The site of convergence of descending efferents and spinal afferents is very likely in the cervical spinal cord. The strongest evidence for this is the increase of spinal excitability after paired stimulation, as indicated by a decrease in spinal threshold. Stimulating the dorsal spinal cord at an intensity above threshold produces a short latency MEP (∼3.5 ms; Figs 4 *C* and 6 *B*). This short response time indicates the response is mediated through spinal circuits, likely segmental connections between afferents and motoneurons. Indeed, there is a striking similarity in the latency of spinal MEPs to the latency of the H‐reflex which we (data not shown) and others have recorded at 3–4 ms (Hosoido *et al*. 2009; Tan *et al*. 2012). This further corroborates segmental changes involving large diameter afferents, which mediate the H‐reflex. The nature of the descending output following cortical stimulation is likely corticospinal because it is a crossed response, and the response abolishes with pyramidal tract section (Sindhurakar *et al*. 2017).

Repetitive paired stimulation caused lasting increases in cortical MEPs and decreases in spinal thresholds for each of the three protocols we tested (Fig. 7). The timing of these changes was strikingly similar for 5 min of paired motor cortex and midline spinal cord stimulation (Fig. 7 *A*). Increasing the duration of stimulation to 10 min (Fig. 7 *B*) or stimulation over the DREZ (Fig. 7 *C*) caused similar decrease in spinal thresholds, but the increases in cortical MEPs was delayed. Both DREZ stimulation and 10 min pairing protocols showed significant elevation in cortical MEPs at 40 min, which was longer than the 5 min midline stimulation protocol.

There are several reasons why the peak MEP effects might be delayed in these protocols, even though the durability of effects was more robust. First, delay in peak excitability has been demonstrated in long term potentiation produced in hippocampus (Otmakhov *et al*. 2004). The complex mechanisms of presynaptic and postsynaptic potentiation take time to develop after the pairing has ended (Feldman, 2012). Also, we (Carmel *et al*. 2010b; Carmel & Martin, 2014) and others (Siebner *et al*. 2004) have observed that responses to cortical stimulation decrease over time, likely through cortical homeostatic mechanisms. Thus, the duration and the site of paired stimulation may need to be balanced to achieve the most robust and durable effects.

Finally, the interactions of spinal afferent stimulation and motor cortex stimulation can be timed to interact in the brain. We provisionally interpret the augmenting effects of spinal cord stimulation 20 ms before motor cortex stimulation (Fig. 2, −20 ms) to be interaction in cortex. These effects may be analogous to the paired associative stimulation protocol in human (Stefan *et al*. 2000), which combines median nerve stimulation with motor cortex stimulation. If this is true, it means that the rat could be a useful model of a popular human plasticity model. It also suggests that the spinal cord may be a more effective site for sensorimotor integration than cortex.

### Comparison with other paired stimulation protocols

The immediate effects of the paired motor cortex and spinal cord protocol only augmented responses, whereas repetitive paired stimulation protocols in primates (Nishimura *et al*. 2013) and humans (Taylor & Martin 2009) also caused depression, depending on the latency of pairing. The ability to modulate responses up or down is a signature of protocols that target the input neuron in motor cortex.

This paired stimulation protocol produces strong and lasting neuromodulation that compares favourably to effect size of modulation observed in previous protocols. We observed an approximate doubling of cortical MEPs after repeated pairing, and this lasted for approximately 60 min. The STDP protocol in non‐human primates using sharp electrodes in motor cortex and the cervical spinal cord and timed for augmentation produced an increase of 6% in evoked responses (Nishimura *et al*. 2013). Pairing TMS and peripheral nerve stimulation in humans produced increases of 33% (Taylor & Martin, 2009) to 40% (Bunday & Perez, 2012). Two paired stimulation protocols were designed to alter leg MEPs. Spinal associative stimulation in humans pairs suprathreshold tibial nerve stimulation with subthreshold motor cortex stimulation – this produces an increase in the size of the H‐reflex of 25% (Cortes *et al*. 2011). Most similar in magnitude to our results are those of spino‐sciatic associative stimulation, in which the L1 spinal cord and the sciatic nerve were stimulated in coordinated fashion. This protocol approximately doubled the amplitude of the MEP, and this effect lasted for over an hour (Ahmed, 2013).

### Application of protocol

Two critical limitations of our approach – the use of invasive brain stimulation and application to anaesthetized animals – are currently being addressed. The use of invasive motor cortex stimulation can be removed in two ways. First, brain stimulation could be applied non‐invasively in humans using transcranial magnetic stimulation. Second, spinal cord stimulation could be triggered by endogenous motor cortex activity – an approach used by several brain–spinal cord prosthetics (Shanechi *et al*. 2014; Sharpe & Jackson, 2014; Capogrosso *et al*. 2016). The second major limitation is that all of the experiments reported here were performed under general anaesthesia. This allowed high precision, particularly in the placement of the spinal cord electrodes, in order to understand the mechanism of the observed plasticity. In order to translate this to the awake, behaving animal, we have developed implantable spinal cord stimulators that enable safe and effective neuromodulation (Pal *et al*. 2016).

Several attributes of this protocol make it suitable for clinical translation. First, in comparison with continuous spinal cord epidural stimulation that has been used for motor recovery in animals and humans with CNS injury, the single pulse of spinal epidural stimulation in our protocol produced slightly larger increases in MEPs. Like other adaptive stimulation protocols, more function can potentially be achieved with less stimulation (Little *et al*. 2013). In addition, spinal cord stimulation is performed below the threshold for evoking movement, similar to clinical spinal epidural stimulation in people. This will help minimize any pain or adverse sensation and permits movement free of stimulation‐induced responses. Finally, the integration of descending motor pathways and large‐diameter afferents is a critical intersection during nervous system development and skill learning; our approach targets a critical endogenous locus for motor control and learning.

Paired activation of descending motor pathways and cervical afferents could be translated to people using several approaches. The most analogous strategy would be to pair epidural stimulation of motor cortex and cervical spinal cord. Each of these sites of epidural stimulation has been shown to be safe in current clinical practice for pain modulation (Grider *et al*. 2016; Lefaucheur, 2016) or to promote motor recovery (Levy *et al*. 2016). Alternatively, the two sites can be stimulated non‐invasively, with magnetic or electrical stimulation applied to the skin. Finally, exogenous stimulation (magnetic or electrical) could be paired with endogenous activity; recording of activity at one site could be used to trigger electrical stimulation at another site, using the closed‐loop neuromodulation (Harel & Carmel, 2016) approach described above. Overall, the results of this study suggest that this approach has potential. The strong effect size, emerging mechanistic understanding, and practicability of this paired stimulation approach make it suitable for testing to strengthen sensorimotor circuits after central nervous system injury.

## Additional information

### Competing interests

None declared.

### Author contributions

A.M.M. and J.B.C. designed the work. A.M.M., A.P. and J.B.C. acquired data. A.M.M., A.P. and J.B.C. analysed data. A.M.M., A.P. and J.B.C. interpreted data. D.G. wrote MATLAB script to analyse data. A.M.M. and J.B.C. wrote the manuscript. A.M.M. and J.B.C. revised the work. The final version of this manuscript was approval by all authors who agree to be accountable for all aspects of the work. All persons designated as authors qualify for authorship, and all those who qualify for authorship are listed.

### Funding

This work was funded by the Travis Roy Foundation (J.B.C. and A.M.M.) and NIH R21EB020318 (J.B.C.).
